# Psychopathology and Quality of Life in Traumatized or Victimized Underage Individuals as Factors for Forensic Multilevel Assessment—A Pilot Investigation

**DOI:** 10.3389/fpsyt.2019.00684

**Published:** 2019-09-18

**Authors:** Sabine Voelkl-Kernstock, Maria Kletecka-Pulker, Anna Felnhofer, Oswald David Kothgassner, Katrin Skala, Brigitte Hansmann, Thomas Wenzel

**Affiliations:** ^1^Department of Child and Adolescent Psychiatry, Medical University of Vienna, Vienna, Austria; ^2^Department for Ethics and Law in Medicine, Medical University of Vienna, Vienna, Austria; ^3^Department of Pediatrics and Adolescent Medicine, Medical University of Vienna, Vienna, Austria; ^4^Department of Psychiatry and Department for Ethics and Law in Medicine, Medical University of Vienna, Vienna, Austria

**Keywords:** forensic assessment, quality of life, posttraumatic stress disorder, children, adolescents, type of trauma

## Abstract

**Background:** Psychological sequels to criminal violence can be long lasting and severe. They are in many countries not sufficiently considered in court cases as an important circumstance that could be used to assess the severity of the crime, also guiding redress, compensation, and rehabilitation of the victim, and—in children—child custody considerations. So far, the focus of forensic assessment has often been limited to diagnostic categories, especially “posttraumatic stress disorder” (PTSD), a diagnosis that presently is subjected to rapidly changing definitions both in and between diagnostic systems. Other indicators such as quality of life (QoL) might be of equal importance as compared to clinical or research diagnostic categories to understand and evaluate the impact of a crime and the amount of help needed and, in the legal context, redress to be asked. Symptoms might differ depending on the crime encountered.

**Objective and Methods:** QoL and general symptom patterns including a PTSD diagnosis were assessed in a group of 10- to 17-year-old minors with (*n* = 33) and without (*n* = 49) PTSD diagnosis who all had experienced sexual abuse, physical abuse, death of a parent, or their parents’ divorce, using standardized diagnostic instruments.

**Results:** PTSD patients reported a significantly lower QoL than non-PTSD controls. Reported symptom patterns with potential impact on life, such as intrusive thoughts, differed between the victims of different crime types, with the highest rates of both intrusive symptoms and combined symptom profile in victims of sexual abuse. Data indicate that the changes between older and present criteria and between DSM and recently published ICD 11 might help identify different groups and symptom profiles.

**Conclusion:** Specific trauma-related symptom profiles integrating the type of crime encountered and its individual impact on QoL may help improve future forensic assessment and guide compensation and rehabilitation plans. Carefully designed studies are now needed to further explore the use and forensic usability of complex indicators and the impact of violence in different forensic settings.

## Introduction

The International Classification of Diseases (ICD-10) defines trauma as “a stressful event or situation (either short- or long-lasting) of an exceptionally threatening or catastrophic nature, which is likely to cause pervasive distress in almost anyone” (1). Trauma in childhood and adolescence often occurs within the family (2) and may include physical, sexual, and emotional abuse; neglect; being separated from parents; as well as witnessing inter-parental violence (3). These traumatic events have a high recurrence, which in its own right may lead to complex traumatization (4, 5) and to the development of posttraumatic stress disorders (PTSDs) in children and adolescents, with lifetime prevalence rates ranging as high as 9% for 13- to 15-year-old adolescents (6).

PTSD constitutes a complex and heterogeneous syndrome that has been shown to encompass up to 79,794 different symptom constellations that meet the criteria for PTSD (7). During the past decades, however, the symptom clusters that were regarded as indicative of PTSD diagnoses in the American Psychiatric Associations DSM model (8, 9) underwent significant changes that were to a similar degree parallel to those seen in the World Health Organization’s International Classification of Diseases, with again marked differences between DSM 5 and ICD-11. This constitutes a challenge also in the effort to provide reliable standards in forensic assessment (10)—an issue to be discussed later in this article. The fact that, in a further step, issues like functioning and quality of life (QoL) must also be considered in diagnostic assessment has gradually been implemented into both of these major diagnostic systems, and partly achieved by integrating the WHO Assessment Schedule.

The fact that, in a further step, resulting issues like functioning and QoL must also be considered in diagnostic assessment has been slowly implemented into both systems and has partly been achieved by integrating the WHO Disability Assessment Schedule 2 (11). The importance of individual symptom profiles, especially of the actual individual QoL that results from symptoms or in the framework of a diagnosis, has so far been neglected and not covered by most studies.

Literature suggests that apart from the severity, extent, and frequency of trauma exposure (12), the probability of developing PTSD may also significantly depend on the type of traumatic event (13–18). Prior studies (6) found that sexual abuse, rape, childhood neglect, physical abuse, as well as attempted suicide had the highest associations with PTSD and subclinical PTSD when compared to other trauma types such as death of a family member, threat of violence, or serious accidents. Among trauma types, sexual abuse seems to be an especially strong and consistent risk factor for the development of PTSD symptoms (19–22).

In addition to the development of PTSD, the type of trauma may also affect other mental health problems or disorders such as depression, withdrawal behavior, anxiety disorders, suicidal attempts, aggression, antisocial behavior, delinquency, peer problems, or substance abuse (14, 15, 17, 21, 23–29).

Apart from personally experiencing a trauma, witnessing a traumatic event may have similarly deleterious repercussions. McCloskey and Walker (14) reported that children who witnessed the death or illness of a significant other were at the greatest risk of developing PTSD, followed by experiencing recurrent domestic abuse as well violent crime. Similarly, Melhem et al. (15) found that children, whose parents died of suicide, an accidental death, or a sudden natural death, showed higher rates of depression and PTSD than non-bereaved controls. In sum, however, studies comparing those two types of traumata—self-experienced vs. witnessed—with regard to their effect on PTSD symptomatology and other mental health problems are still scarce.

Lastly, a traumatic experience may not only lead to specific psychopathological symptoms, but also have a more general impact on the person’s social ties, friendship, and interests and, thus, may affect the patients’ overall QoL. Past studies (30) substantiate this assumption, showing that posttraumatic stress is significantly linked to a reduction of QoL in children and adolescents. Also, prior findings highlight the negative long-term effects childhood traumata can have on physical, psychological, and social QoL in adulthood (31, 32). Only one study has, however, thus far examined this relationship and found that neglect and sexual abuse were associated with reduced mental health-related QoL, whereas psychological and physical abuse caused a reduction in both mental and physical health-related QoL (33).

To date, research has mainly focused on differences in psychopathology, and especially on the prevalence of clinical symptoms or diagnosis, but not on their specific impact on life in connection with the experience encountered by the victim. Thus, the current study aimed to explore whether 1) experiencing different kinds of traumatic events and 2) fulfilling or not the criteria for a PTSD diagnosis have an impact on symptoms profile and QoL, which should also be considered in civil and criminal court.

## Method

The current study was carried out in a group of consecutive patients at the Trauma and Forensics outpatient clinic at the Department of Child and Adolescence Psychiatry at the Medical University of Vienna. Following a detailed briefing, all parents signed an informed consent form agreeing to the voluntary participation of their children. Minors aged 14 and above also signed informed consent. Accordingly, children and adolescents were provided with written informed consent forms stating that withdrawal would not affect their treatment and that all communications would be anonymous. The local institutional review board approved the study in its current form. All participants were interviewed by expert interviewers (trained psychologists with a long-standing expertise in psychotraumatology). Parents were interviewed separately. The study was approved by the Ethics Committee of the Medical University of Vienna (EK-number: 476/2004).

### Participants

In total, 82 patients (46 female, 36 male) between 10 years 7 months and 17 years 11 months (M = 13.6; SD = 2.348) completed the required questionnaires. All patients had experienced or witnessed a traumatic event, either sexual abuse (*n* = 19), physical abuse (*n* = 19), loss of a close family member (*n* = 19), or the divorce of their parents (*n* = 25). Patients were assessed a month post-trauma at the earliest and not later than 2–3 months after the last traumatic experience. Patients without sufficient language skills or with other psychiatric diagnosis prior to the traumatic event were excluded from the study.

Trauma types covered a range of multiple specific traumatic experiences: 1) Sexual assaults comprised anal/vaginal rape, brutal insertion of fingers or objects, simultaneous abuse by different perpetrators, oral sex, forced masturbation, kissing, and touching (including anal/vaginal area or penis). 2) Physical abuse was defined here as beating in the face or head, knocking unconscious, pulling hairs until bleeding, beating leading to injury, cold/hot showering, and kicks against the body. 3) The loss of a family member included the death of a sibling, mother, or father after an accident, terminal disease, homicide, suicide, or a family member who went missing. 4) The category divorce, in turn, was defined as the divorce of biological parents including legal action, separation from one parent or both parents for an undefined longer period, or the child being a witness of emotional violence between parents or being treated as a property in the conflict situation.

### Measures

ICD-10- and DSM-IV TR-based instruments were used given that these were the only available ones, validated in German, at the time the study was carried out.

**PTSD symptomatology.** The revised form of the Impact of Event Scale-Revised (IES-R) (34) was used to assess subjective distress caused by traumatic events. The 22-item self-report measure was completed by the participating children and adolescents. It examines symptoms of intrusive thoughts (criterion B), avoidance (criterion C), and hyperarousal (criterion D). The IES-R is a valid and reliable screening instrument for PTSD and has previously been used in several studies investigating PTSD symptomatology (35–37). The IES-R shows a good overall reliability (α = 0.93; scales α range from 0.83 to 0.89). In addition, a routine screening checklist for PTSD symptoms developed locally for everyday clinical documentation independent from the research project was given to the participants as they were also offered treatment in our outpatient department.

**General symptomatology.** The school-age assessment forms of the Child Behavior Checklist (CBCL 6–18) (38) are based on DSM-IV categories and represent an empirically based screening of the following psychopathological dimensions: (39) socially withdrawn, (40) somatic complaints, (41) anxious/depressed, (42) social problems, (43) thought problems, (44) attention problems, as well as (45) rule-breaking behavior and (46) aggressive behavior. The CBCL 6–18 was completed by children’s biological mothers (*n* = 57), biological fathers (*n* = 17), stepmothers (*n* = 2), and a grandmother (*n* = 1). Five parents did not complete this questionnaire.

The German version of the Structured Clinical Interview for DSM-IV (SCID) PTSD and Major Depression section (47) was administered by trained psychologists to diagnose PTSD and differentiate PTSD and depression or acute stress reactions in children and adolescents.

**Quality of life.** The Inventory for the Assessment of Quality of Life in Children and Adolescents (ILK) (48) is a disorder-nonspecific screening instrument that assesses QoL in children with and without mental disorders on the following items: school, family, peer contacts, interests, and free time, as well as stress through assessment and therapy and stress through the mental disorder or, somatic disease, as well as an overall rating of the patients’ QoL. The ILK shows an adequate overall reliability of α = 0.62 in the current study. It has been widely used in a number of studies with different clinical populations and has been shown to be to be a useful and reliable instrument (39–46).

## Results

ANOVAs were computed to analyze differences between trauma types regarding PTSD symptomatology, behavioral symptomatology, and QoL. Statistical analyses were carried out using SPSS 20 (IBM) and considering an alpha error of 5% in all calculations. Below, the results are presented according to the abovementioned research questions.

Post-assessment, 33 patients were diagnosed with PTSD (ICD-10-Code F43.1), whereas 49 patients did not fulfill the criteria for a PTSD diagnosis. **Table 1** shows the frequency of comorbid disorders in PTSD and non-PTSD children after traumatic events.

**Table 1:** Comorbid psychiatric disorders in non-PTSD and PTSD sample (n = 82).

	Non-PTSD		PTSD	
	n = 49		n = 33	
	n	% within group	n	% within group
ADHD	6	12.2%	6	18.2%
Anxiety	12	24.5%	7	21.2%
Conduct Disorder	1	2.0%	1	3.0%
Depression	6	12.2%	5	15.2%
Enuresis	–		2	6.1%
OCD	2	4.1%	–	
Substance Abuse	–		1	3.0%
Total	27	55.1%	22	66.7%

OCD, obsessive–compulsive disorder.

### PTSD Symptomatology

**Figure 1:**
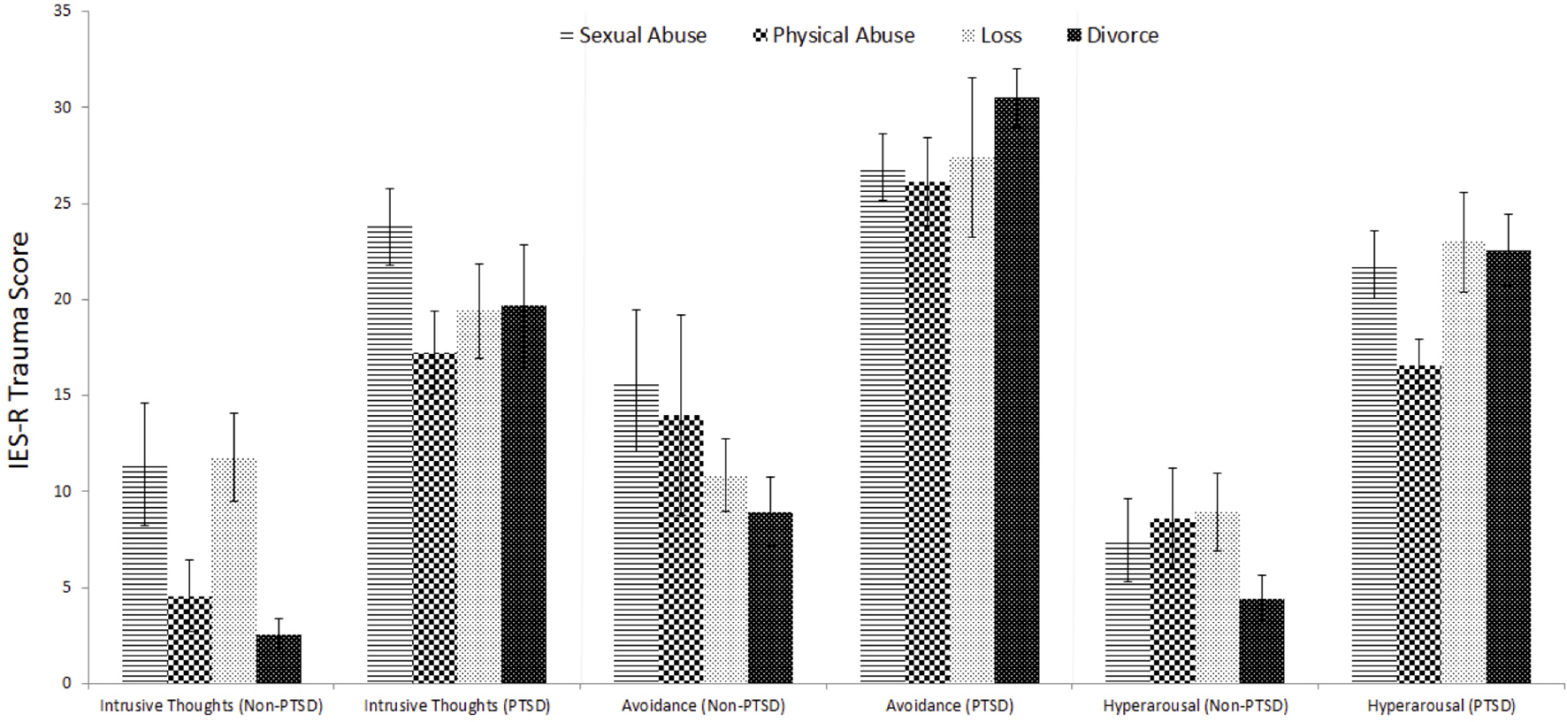
PTSD-specific symptomatology assessed by IES-R (*n* = 82).

**Figure 1** shows differences between the four trauma-type groups in means and the standard error of the mean for the key PTSD symptoms intrusive thoughts, avoidance, and hyperarousal. More specifically, the two-factor ANOVA with Bonferroni *post hoc* tests indicated a significant main effect of trauma type for intrusive thoughts (*F*_3,74_ = 4.260; *p* = 0.008; par. η² = 0.147) with children reporting significantly less intrusive thoughts after their parents’ divorce than those children who experienced sexual abuse (*p* < 0.001), physical abuse (*p* = 0.035), or loss of a significant other (*p* = 0.006). Additionally, there was a significant difference between children with PTSD and without PTSD (*F*_1,74_ = 56.421; *p* < 0.001; par. η² = 0.433) regarding intrusive thoughts or flashbacks. With regard to avoidance, there was no significant difference between the four traumatic events (*F*_3,74_ = 0.214; *p* = 0.887; par. η² = 0.009), but there was a significantly relevant difference between children with and without PTSD (*F*_1,74_ = 57.192; *p* < 0.001; par. η² = 0.436). Similarly, no difference in hyperarousal was found between the trauma types (*F*_3,74_ = 1.547; *p* = 0.210; par. η² = 0.059), but again a significant main effect emerged for PTSD diagnosis (*F*_1,74_ = 104.287; *p* < 0.001; par. η² = 0.585). **Table 2** shows the descriptive results of the symptom screening using an interview form.

**Table 2:** Symptom screening checklist interview with patients (n = 78).

		Sexual abuse	Physical abuse	Loss	Divorce
*Somatization*	PTSD	60%	33%	60%	0%
	Control	67%	43%	31%	39%
*Rapid performance loss*	PTSD	100%	58%	100%	75%
	Control	66%	57%	77%	67%
*Sleeping problems*	PTSD	70%	75%	40%	25%
	Control	33%	57%	25%	33%
*Irritability*	PTSD	90%	75%	100%	75%
	Control	78%	71%	69%	83%
*Anger*	PTSD	90%	66%	100%	50%
	Control	78%	71%	69%	72%
*Attention deficit*	PTSD	90%	83%	80%	100%
	Control	100%	71%	85%	89%
*Alertness*	PTSD	80%	50%	60%	50%
	Control	44%	43%	53%	55%
*Startle response*	PTSD	70%	58%	40%	75%
	Control	44%	57%	53%	56%
*Amnesia*	PTSD	10%	8%	0%	0%
	Control	0%	0%	0%	5%
*Avoidance*	PTSD	90%	50%	60%	100%
	Control	77%	85%	69%	67%
*Social withdrawal*	PTSD	70%	42%	80%	50%
	Control	55%	100%	54%	67%
*Alienation*	PTSD	50%	58%	60%	100%
	Control	44%	71%	23%	50%
*Reduced facial expressions*	PTSD	60%	33%	80%	0%
	Control	22%	28%	23%	28%
*Hopelessness*	PTSD	30%	41%	20%	0%
	Control	22%	29%	23%	23%
*Flashbacks*	PTSD	50%	41%	40%	25%
	Control	22%	29%	31%	22%
*Nightmares*	PTSD	60%	17%	0%	50%
	Control	33%	29%	23%	44%

PTSD, children with a PTSD diagnosis (n = 33); Control, children without PTSD diagnosis (n = 45), n = 4 patients did not respond to the interview form.

### Behavioral Symptomatology

According to the CBCL scores of *n* = 78 parents (of children with PTSD diagnosis, *n* = 33; without a PTSD diagnosis, *n* = 49), a statistically relevant difference between the four types of traumatic events was found for *social withdrawal* (*F*_3,68_ = 3.261; *p* = 0.027; par. η² = 0.126). After Bonferroni correction, results indicated a significant difference in social withdrawal between children after sexual trauma and children after loss or divorce (*p* < 0.05). With an alpha error increase to 10%, differences between these two subgroups and children after physical abuse also became significant. Furthermore, an interaction effect of types of traumatic events and the groups of children with or without PTSD was found (*F*_3,68_ = 3.005; *p* = 0.036; par. η² = 0.117), indicating that, in particular, children with PTSD after sexual abuse show the highest levels of social withdrawal.

With regard to *somatic complaints*, children whose parents were divorced reported less complaints than children who experienced sexual abuse (*F*_3,68_ = 5.342; *p* = 0.002; par. η² = 0.191). Also, there was a significant main effect of trauma type regarding *anxious and depressive behaviors* (*F*_3,68_ = 3.670; *p* = 0.016; par. η² = 0.139); corresponding Bonferroni analyses revealed a difference between children after sexual abuse and children after the loss of a family member (*p* = 0.032) and those who experienced the divorce of their parents (*p* = 0.002).

Regarding *thought problems*, again a significant effect was found for the type of traumatic event (*F*_3,68_ = 3.670; *p* = 0.017; par. η² = 0.138). Bonferroni analyses indicated group differences between sexually abused and physically abused children (*p* = 0.027) as well as children who experienced loss (0.026), but not those with divorced parents (*p* = 1.000). A significant interaction effect showed that children with PTSD particularly tended to have more thought problems after sexual trauma or divorce (*F*_3,68_ = 4.419; *p* = 0.007; par. η² = 0.163).

**Figure 2:**
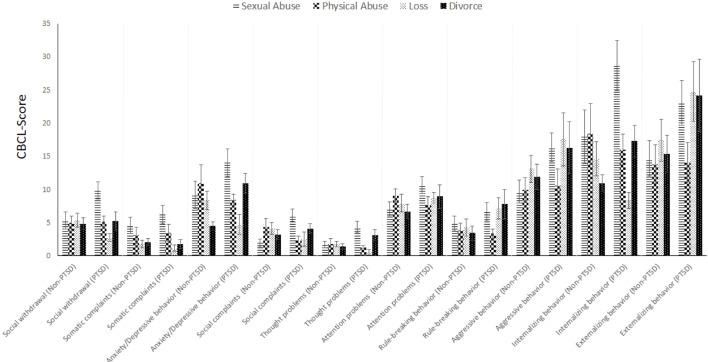
General symptomatology assessed by parental judgment (*n* = 78).

Finally, differences between children with and without PTSD were found regarding *rule-breaking behavior* (*F*_3,68_ = 4.827; *p* = 0.031; par. η² = 0.066) and *aggressive behavior* (*F*_3,68_ = 4.916; *p* = 0.020; par. η² = 0.067), showing that children with a PTSD diagnosis tend to show more oppositional behavior than children who experienced a traumatic event but were not diagnosed with PTSD. Interestingly, there were no significant differences for *social complaints* or *attention problems*, either between children with and without PTSD or between the four trauma types. For an overview over general symptomatology, see **Figure 2**.

### QoL

**Figure 3:**
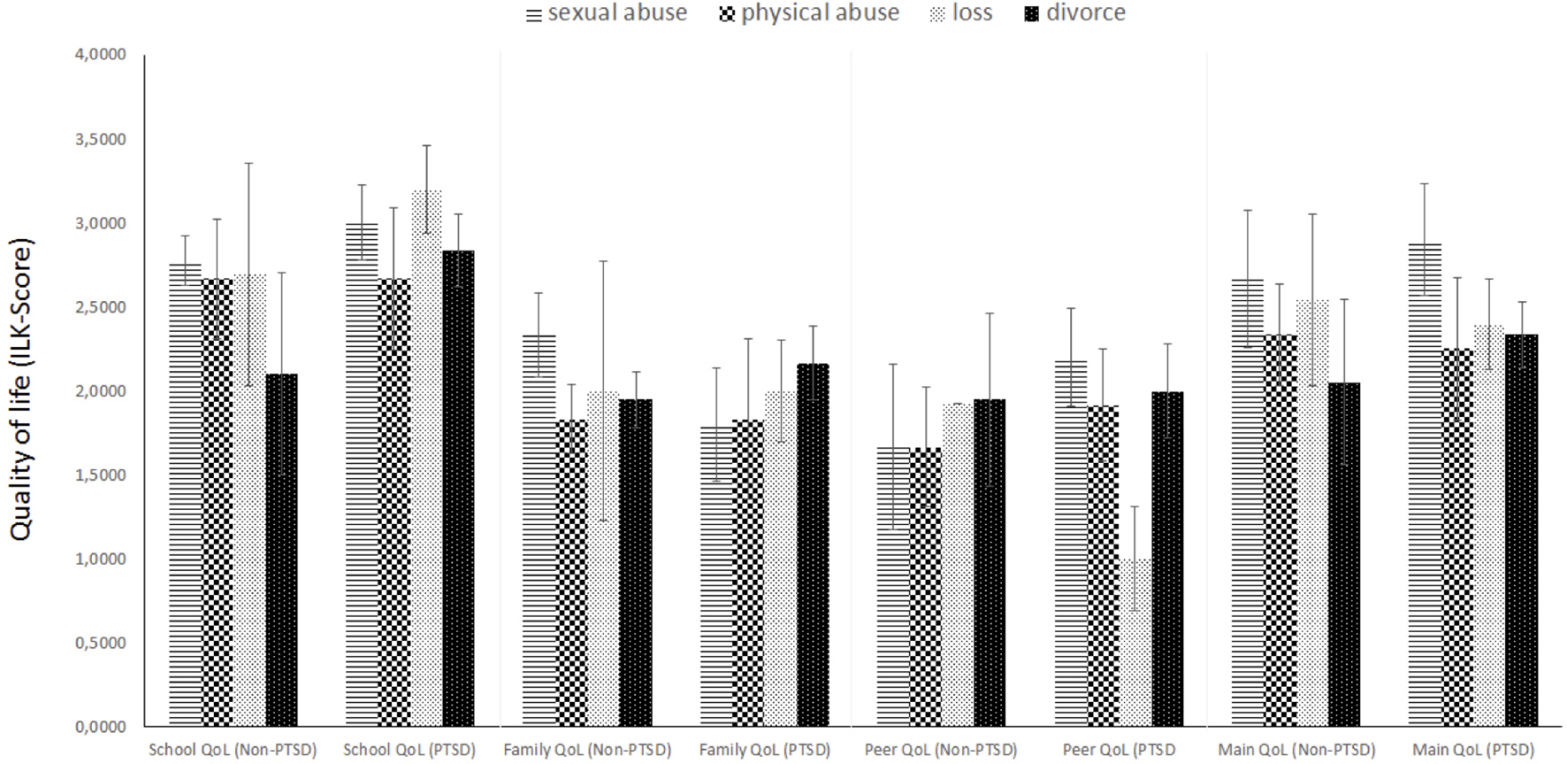
Quality of life assessment measured by ILK (*n* = 80).

There was no difference in children’s QoL between the four different types of traumatic events; however, children with and without a PTSD diagnosis reported a significantly different QoL. Children with PTSD especially rated stressors related to the mental disorder as significantly higher than their non-PTSD diagnosed peers (*F*_3,68_ = 4.092; *p* = 0.047; par. η² = 0.054); in contrast, there were no group differences regarding stressors related to assessment or therapy. Moreover, children with PTSD described significantly more problems with their mental health (*F*_3,72_ = 9.698; *p* = 0.003; par. η² = 0.119). As shown in **Figure 3**, no group differences (either for trauma type or for PTSD diagnosis) were found regarding school, family, peer contacts, and main QoL assessment.

## Discussion

The main objective of the study was to assess whether children and adolescents respond by different symptom patterns to four types of traumatic events and to assess QoL in both samples of PTSD and non-PTSD children and adolescents.

### PTSD Symptomatology

First of all, the three DSM-IV symptom clusters discriminated well between the PTSD and non-PTSD groups, with PTSD subjects showing significantly more intrusive thoughts (criterion B), avoidance (criterion C), and hyperarousal (criterion D). When comparing the four trauma types, no group differences were found except for intrusion. Children and adolescents who experienced sexual abuse reported substantially more intrusive symptoms than children with a history of other traumatic experience regardless of whether those subjects were subsequently diagnosed with PTSD or not. Generally, the experience of sexual abuse showed the most consistent negative effects on most relevant symptom scales. For instance, sexual abuse victims reported most somatic complaints, most anxious and depressive symptoms, as well as thought problems, and they showed strongest social withdrawal when compared to the other groups. Additionally, children who were both diagnosed with PTSD and had a history of sexual assault were at double jeopardy of developing thought problems and being socially withdrawn. These results are generally in line with prior studies (19–21, 22, 49) and once again emphasize the particularly deleterious effects of sexual abuse on a child’s mental health.

In particular, the significantly heightened level of intrusive thoughts and thought problems in sexual abuse victims is a consistent finding across past studies (50).

### PTSD Criteria in DSM 5

The current study was, as noted earlier, based on the DSM-IV criteria for PTSD as no appropriate, validated German DSM 5 assessment tools existed at the time of the study’s realization. Yet, in comparison to the DSM-IV, the DSM 5 (9) released in 2013 now entails 20 instead of 17 symptoms and four instead of the initial three symptom clusters. The avoidance criterion C was separated into two clusters, avoidance (criterion C) and negative alterations in cognitions and mood (criterion D).

First studies (51, 52) involving children aged 7–11 years reveal differences in PTSD diagnosis and low agreement between the two diagnostic systems. Also, the DSM 5 had low endorsement rates and identified fewer children than the three-cluster model in DSM-IV; in particular, the cognitions/mood and the avoidance clusters were found to preclude many children (51). The authors conclude that the nature of these clusters, and especially their emphasis on sophisticated cognition processes and internalization, may be inappropriate for preadolescent children. Similarly, the best model fit for children and adolescents was found for the three-cluster PTSD model proposed by ICD-11 (52) as opposed to the four-cluster model of the DSM 5 (53). Future studies should, thus, make a conscious effort to incorporate a developmental perspective that is sensitive to age-specific cognitive and emotional maturation processes (54). Given furthermore the trauma type specific symptom clusters found in this study, it is possible that symptom severity and presentation may vary as a function of not only age but also trauma type. Additional research is needed to further debunk these associations and shed more light onto the complex associations between type of trauma, age, and symptom presentation.

### Crime Type-Related Symptom Patterns and QoL

When disentangling the differential effects of specific trauma types, very distinct symptom profiles for four trauma types can be found. Sexual abuse predominantly evokes internalizing symptoms such as social withdrawal and social problems as well as anxiety and depression. Also, children with a history of sexual abuse report more somatic complaints when compared to those who experienced a divorce or the loss of a parent. Depression and anxiety, in turn, are elevated especially in children who witnessed a divorce and subsequently develop PTSD symptoms. Finally, the afflicted tend to more frequently show oppositional or aggressive behavior, which may also result in delinquency.

The profiles found here correspond partly with results from other studies; yet, comparisons are difficult because, to our knowledge, no study has thus far directly compared the four trauma types considered here. Similar studies, however, found victims of sexual abuse to report above average overall PTSD cluster symptoms (avoidance, intrusion, and hyperarousal) but also to show more depressive and internalizing symptoms than victims of physical abuse (55). These specific symptom profiles may be used to inform forensic assessment as well as customized treatment for PTSD patients with different histories of traumatic experiences. For instance, the most afflicted group of sexual abuse victims may especially profit from high-frequency trauma-focused cognitive behavioral therapy with an emphasis on trauma narrative, cognitive coping, and trauma processing skills with a focus not only on PTSD symptoms but also on comorbid mental health problems (56).

Finally, results concerning QoL indicate that, while all four groups reported an equally low QoL, a PTSD diagnosis seems to adversely affect overall QoL. The significant difference in self-reported stressors relating to the mental disorder as well as mental health problems between PTSD patients and non-PTSD patients suggests a strong effect of PTSD symptoms alone on QoL. Comorbid disorders may be partialled out as both groups showed comparable comorbidities. Hence, children and adolescents are at a heightened risk of suffering from multiple drawbacks in many areas of life, a result that is in line with prior research (30).

### Limitations of This Study and Considerations for Future Research

Our study was built solely on a sample of help-seeking, clinical clients. Future studies should also use healthy controls as a basis for further debunking the etiology of PTSD in children and adolescents (14). Additionally, gender could be taken up as a possible risk factor of PTSD symptom development (18). In the current study, gender was equally distributed across the groups; however, no conclusions can be drawn regarding the differential effect of trauma type on gender or gender-specific differences in symptom profiles. Also, we could not include further specific trauma features besides type of violence encountered such as frequency. This will undoubtedly pose a challenge for future research, especially when attempting to control for the confounding influence of multiple stressors often encountered in families with low socioeconomic status and a history of violence. At the same time, however, the choice to use the non-PTSD group of children and adolescents as controls helped to control for the influence of a traumatic experience, since all participants had a history of trauma. Also, it allowed for direct comparisons and for partialling out the mediating effect of comorbid disorders on QoL or on associated mental health problems.

## Conclusion

We succeeded in showing that there is a distinct impact of trauma type on overall psychopathology with sexual abuse entailing the most debilitating consequences including, in particular, QoL for the affected individual. Also, PTSD symptoms in themselves have a wide-ranging effect on multiple areas of life, significantly diminishing the overall QoL of patients, indicating that the development of this disorder might be the most important predictor of low QoL. In assessment, care should be taken not to limit assessment of PTSD symptoms and presence or absence of clinical diagnosis, as well as to evaluate individual symptom patterns and their impact on QoL to better elucidate the specific impact of violent crimes and abuse.

A special challenge in different settings of forensic assessment will also be set by the marked differences between criteria using the different APA and WHO systems and their recent revisions that are based on highly diverse symptom clusters and different categorical models. This must be discussed when considering evaluation practices and standards. QoL or social functioning indicators and a better understanding of individual symptom profiles and their impact on everyday life might be a better means to understand impact and consequent needs for courts dealing with violence and abuse.

Further studies exploring the use of the different diagnostic systems, daily functioning, and QoL would be desirable.

## Author Contributions

SV-K was the lead researcher and coordinator of the study. MK-P contributed as the main legal and victimology expert. AF, KS, and BH have assisted in the development of the project, contacted patients, and collected and inserted the data. OK contributed to data analysis. TW contributed in supervision, writing, data analysis, and final submission.

## Funding

This study has been performed by means of the “Bürgermeister-Fonds” of the city of Vienna (Number: 2421).

## Conflict of Interest Statement

The authors declare that the research was conducted in the absence of any commercial or financial relationships that could be construed as a potential conflict of interest.
